# Activation of farnesoid X receptor suppresses ER stress and inflammation *via* the YY1/NCK1/PERK pathway in large yellow croaker (*Larimichthys crocea*)

**DOI:** 10.3389/fnut.2022.1024631

**Published:** 2022-11-24

**Authors:** Jianlong Du, Junzhi Zhang, Xiaojun Xiang, Dan Xu, Kun Cui, Kangsen Mai, Qinghui Ai

**Affiliations:** ^1^Key Laboratory of Aquaculture Nutrition and Feed (Ministry of Agriculture and Rural Affairs), The Key Laboratory of Mariculture (Ministry of Education), Ocean University of China, Qingdao, China; ^2^Laboratory for Marine Fisheries Science and Food Production Processes, Qingdao National Laboratory for Marine Science and Technology, Qingdao, China; ^3^Laboratory for Marine Fisheries Science and Food Production Processes, Qingdao National Laboratory for Marine Science and Technology, Qingdao, China

**Keywords:** FXR, ER stress, inflammation, large yellow croaker, PERK

## Abstract

Unfolded protein responses from endoplasmic reticulum (ER) stress have been implicated in inflammatory signaling. The vicious cycle of ER stress and inflammation makes regulation even more difficult. This study examined effects of farnesoid X receptor (FXR) in ER-stress regulation in large yellow croakers. The soybean-oil-diet-induced expression of ER stress markers was decreased in fish with FXR activated. In croaker macrophages, FXR activation or overexpression significantly reduced inflammation and ER stress caused by tunicamycin (TM), which was exacerbated by FXR knockdown. Further investigation showed that the TM-induced phosphorylation of PERK and EIF2α was inhibited by the overexpression of croaker FXR, and it was increased by FXR knockdown. Croaker NCK1 was then confirmed to be a regulator of PERK, and its expression in macrophages is increased by FXR overexpression and decreased by FXR knockdown. The promoter activity of croaker NCK1 was inhibited by yin-yang 1 (YY1). Furthermore, the results show that croaker FXR overexpression could suppress the P65-induced promoter activity of YY1 in HEK293t cells and decrease the TM-induced expression of *yy1* in macrophages. These results indicate that FXR could suppress P65-induced *yy1* expression and then increase NCK1 expression, thereby inhibiting the PERK pathway. This study may benefit the understanding of ER stress regulation in fish, demonstrating that FXR can be used in large yellow croakers as an effective target for regulating ER stress and inflammation.

## Introduction

Inflammation occurs when the body responds to harmful stimuli (1). Poor diets such as excessive fat, unbalanced nutrition and specific nutrient deficiency, are important factors that promote chronic inflammation that can last for months or years (2–5). The replacement of fish oil with vegetable oil (VO) has become increasingly common in aquaculture. However, the excessive use of vegetable oil has been shown to alter the metabolic balance and induce inflammation in fish, causing growth inhibition and health issues (6–9). Thus, there is an urgent need to develop strategies to regulate inflammation in fish. The endoplasmic reticulum (ER) is crucial for cell housekeeping (10). Under stress, the ER becomes stressed from the accumulation of misfolded and unfolded proteins, resulting in the unfolded protein response (UPR) (11). Mammals (12, 13) and fish (14, 15) have both been shown to develop inflammation when exposed to chronic and excessive ER stress. It has been shown that the UPR pathway plays an important role in inflammation (10–13), and some studies have shown that inflammatory stimuli induce ER stress (16, 17). In many diseases, inflammation and ER stress occur at the same time. The interplay between ER stress and inflammation could cause a vicious circle that may worsen the disease (18, 19). As a result, there is a need to explore targets that would regulate ER stress, which may be helpful to break the vicious cycle and alleviate inflammation in fish.

Farnesoid X receptor (NR1H4) is a transcription factor regulating a variety of genes that are involved in bile acid metabolism, lipid metabolism, glucose metabolism and inflammation (20). As a member of the nuclear receptors, FXR can be activated or suppressed by ligands, making it a potential target for exogenous regulation (20, 21). However, only a small amount of research on FXR in fish has been conducted. Our previous studies showed that the FXR is highly expressed in the liver, kidney and intestine of large yellow croakers (*Larimichthys crocea*) (8), and GW4064 and chenodeoxycholic acid (CDCA) could effectively activate it (22). Furthermore, in croakers, FXR is also implicated in lipid metabolism and inflammation (22, 23). Our previous study showed that FXR had anti-inflammatory properties in croakers by directly and indirectly suppressing the activity of NFκB-P65 activity. Previous studies in mammals found that FXR interacted with endoplasmic reticulum stress (24, 25). When ER stress was activated in aging mice, the expression of FXR in the liver decreased (25). Our previous study showed that ER stress also reduced FXR expression in fish fed a high-lipid diet (23). A study in mice showed that FXR activation could suppress the ER-stress-induced expression of NLRP3 *via* Non-catalytic region of tyrosine kinase adaptor protein 1 (NCK1) (24). However, the role of FXR and NCK1 in regulating ER stress and how FXR activation affects NCK1 expression in fish is unknown.

Large yellow croaker is widely cultured in China and is of high economic value. In previous studies, large yellow croakers showed frequent inflammation induced by VO diets (22, 26, 27). A macrophage cell line for large yellow croakers has been well established (28). Based on this research, large yellow croaker is an appropriate model for immunological studies (28–30). This study focused on understanding the role of FXR in regulating ER stress and inflammation and the underlying mechanisms which could pinpoint a potential target for breaking the cycle of ER stress and inflammation in fish.

## Methods

### Feeding experiment

As described previously, an animal feeding experiment was conducted (31). In brief, Fish oil (FO) and soybean oil (SO) diets, as well as soybean oil (SO) diets that contain CDCA (C9377, Sigma) at 300 mg or 900 mg/kg (SO-CDCA 300 or SO-CDCA 900) were developed (31) (Supplementary Table 1). Randomly, each diet was assigned to a cage (60 fish/cage, 10.03 ± 0.02 g) in triplicate. Fish were fed two times a day at 05:00 and 17:00 until satiation with under appropriate conditions for 10 weeks. After fasting for 24 hours, the fish were anesthetized with eugenol (1:10,000) at the end of the feeding trial. Then, six individuals in one cage were sampled for head kidney tissues and immediately frozen in liquid nitrogen.

### Cell culture and treatment

Croaker macrophages were cultured as previously described (27°C and Dulbecco's Modified Eagle Medium: Nutrient Mixture F-12 with 15% fetal bovine serum) (28). HEK 293T cells were cultured as previously described (5% CO_2_, 37°C, and Dulbecco's Modified Eagle Medium with 10% fetal bovine serum) (23). To explore the role of FXR in ER stress regulation, we established macrophages stably transfected for FXR overexpression or knockdown. Macrophages were stably transfected with a recombinant lentivirus encoding the croaker FXR (LvFXR) and a lentivirus-based shRNA vector targeting FXR (siFXR). Macrophages infected with a negative-control lentivirus were used as a control. The cells were treated with lentivirus for 48 h before they were screened with puromycin in gradient concentrations until they could continue to grow and pass on.

To investigate the effect of LA on ER stress, macrophages were treated with linoleic acid (LA, 100 μm) for 0, 0.5, 1, 2, 4, and 6 hours or treated with LA (0, 100, 200, and 400 μm) for 4 hours. To confirm the effect of activation of ER stress on inflammation, macrophages were treated with an ER stress inducer tunicamycin (TM, 4 μm, MCE) for 0, 0.5, 1, 2, 4, and 6 hours. In order to determine whether the FXR regulates ER stress and inflammation, macrophages were pre-treated with FXR agonists CDCA (50μm, Sigma) and GW4064 (2μm, MCE) for 1 h and then stimulated with TM (4 μm) for 4 hours. To find out how FXR affected ER stress, the control cells, LvFXR cells, and siFXR cells were treated with 4 μM TM for 4 h. To confirm the effect of NCK1 on PERK of large yellow croaker, HKE 293T cells were co-transfected with croaker NCK1 and PERK-GFP plasmids and then stimulated with TM (4 μm) for 4 h.

### QRT-PCR

Real-time quantitative PCR was used to determine gene mRNA expression as described by Du et al. (31). The mRNA level of activating transcription factor 4 (ATF4), ATF6, cyclooxygenase 2 (COX2), IL6, NCK1, interleukin 1 beta (IL1β), C/EBP homologous protein (CHOP), tumor necrosis factor alpha (TNFα), glucose-regulated protein 78 (GRP78), eukaryotic initiation factor 2α (EIF2α), yin-yang 1 (YY1) and X-box-binding protein 1s (XBP1s) was detected (Supplementary Table 2). Housekeeping genes were β-actin and GAPDH. A normalized gene expression level was calculated and normalized using the the 2^−Δ*ΔCT*^ method (32).

### Western blotting

According to Tan et al., western blots were performed (26). In the present study, antibodies against FXR (Bioss, China), HA (CST, USA), GRP78 (CST, USA), GAPDH (ZSGB-Bio, China), p-PERK (Bioss, China), PERK (CST, USA), p-EIF2α (CST, USA), XBP1s (CST, USA), and ATF6 (Bioss, China) were used.

### Luciferase reporter assay

Croaker NCK1 and YY1 promoters were cloned into a pGL3-basic reporter vector (Promega, USA). The plasmids expressing croaker FXR, small heterodimer partner (SHP), P65, PERK-GFP, NCK1, and YY1 were stored in our laboratory. To explore the effect of YY1 on NCK1 promoter of large yellow croaker, HKE 293T cells were co-transfected with reporter plasmids (NCK1 promoter), phRL-CMV plasmid, and expression plasmid (croaker YY1) for 24 h. To investigate the effect of FXR and SHP on the activity of YY1 promoter, HKE 293T cells were co-transfected with reporter plasmids (YY1 promoter), phRL-CMV plasmid, and expression plasmid (croaker P65, FXR or SHP) for 24 h. Following the previous description, cell transfection and fluorescence detection were performed (22, 23).

### Statistical analysis

The data (means ± SEMs.) were analyzed with SPSS 20.0 (SPSS, USA), utilizing ANOVA and Duncan's multiple-range test for significance. The difference between the two groups was tested with Student's *t*-tests. Statistical significance was determined by *P* < 0.05.

## Results

### CDCA supplementation suppressed SO-diet-induced ER stress *in vivo*

**Figure 1:**
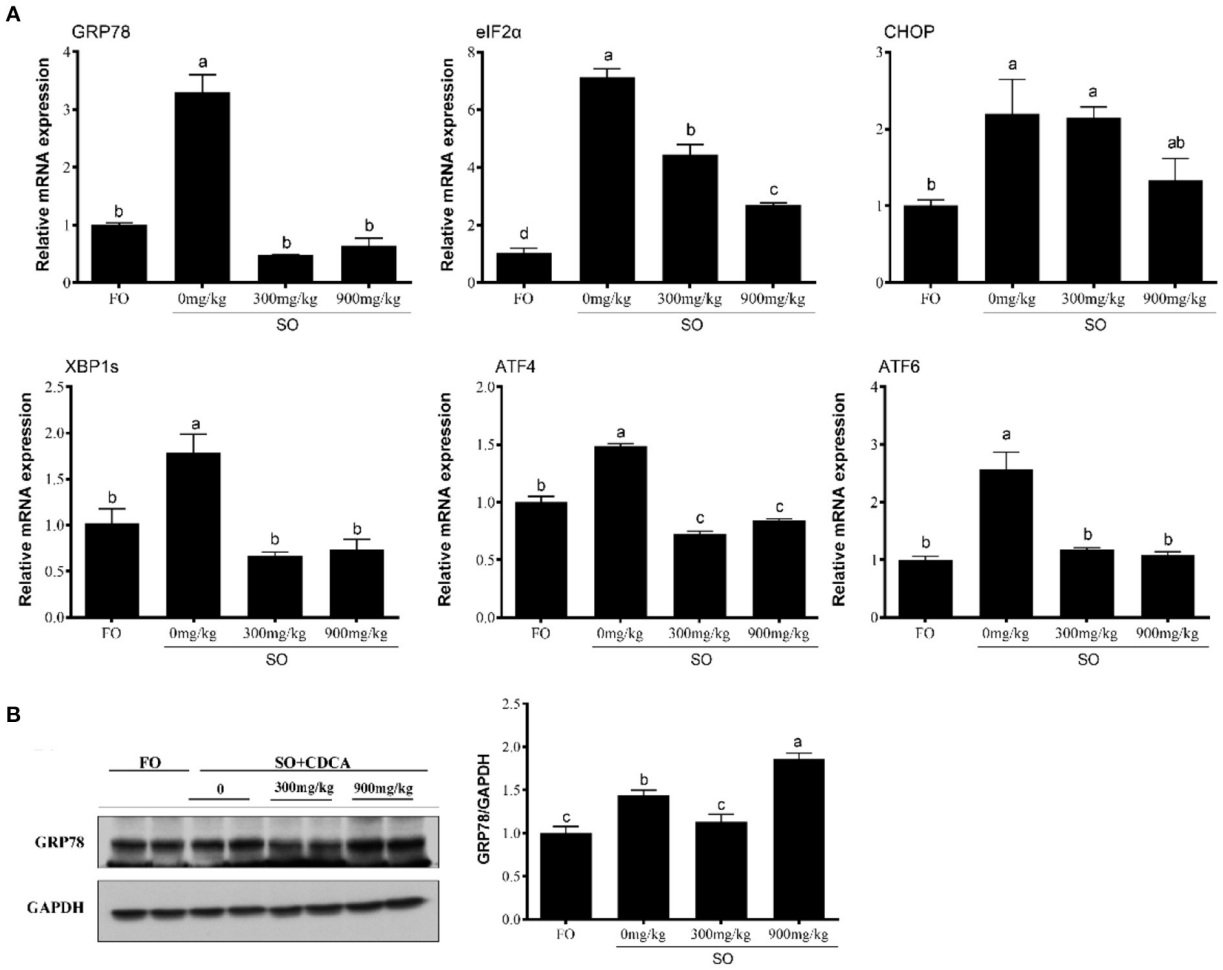
Expression of **(A)** ER stress-related genes and **(B)** GRP78 protein levels in the head kidney of fish after SO feeding and CDCA supplementation. The significance was analyzed by one-factor ANOVA and Duncan's Multiple Range Test (means ± SEMs; *n* = 3). Means without the same label were considered different, *P* < 0.05.

Compared to the FO group, the SO diet significantly increased gene expression associated with ER stress, such as GRP78, EIF2α, CHOP, XBP1S, ATF4, and ATF6 (*P* < 0.05) (Figure 1A). CDCA supplementation significantly decreased the SO-diet-induced mRNA level of GRP78, EIF2α, CHOP, XBP1S, ATF4, and ATF6 (*P* < 0.05) (Figure 1A). As well, the SO-CDCA300 group had a lower level of GRP78 protein than the SO group (*P* < 0.05) (Figure 1B).

### Effects of LA treatment on ER stress in croaker macrophages

**Figure 2:**
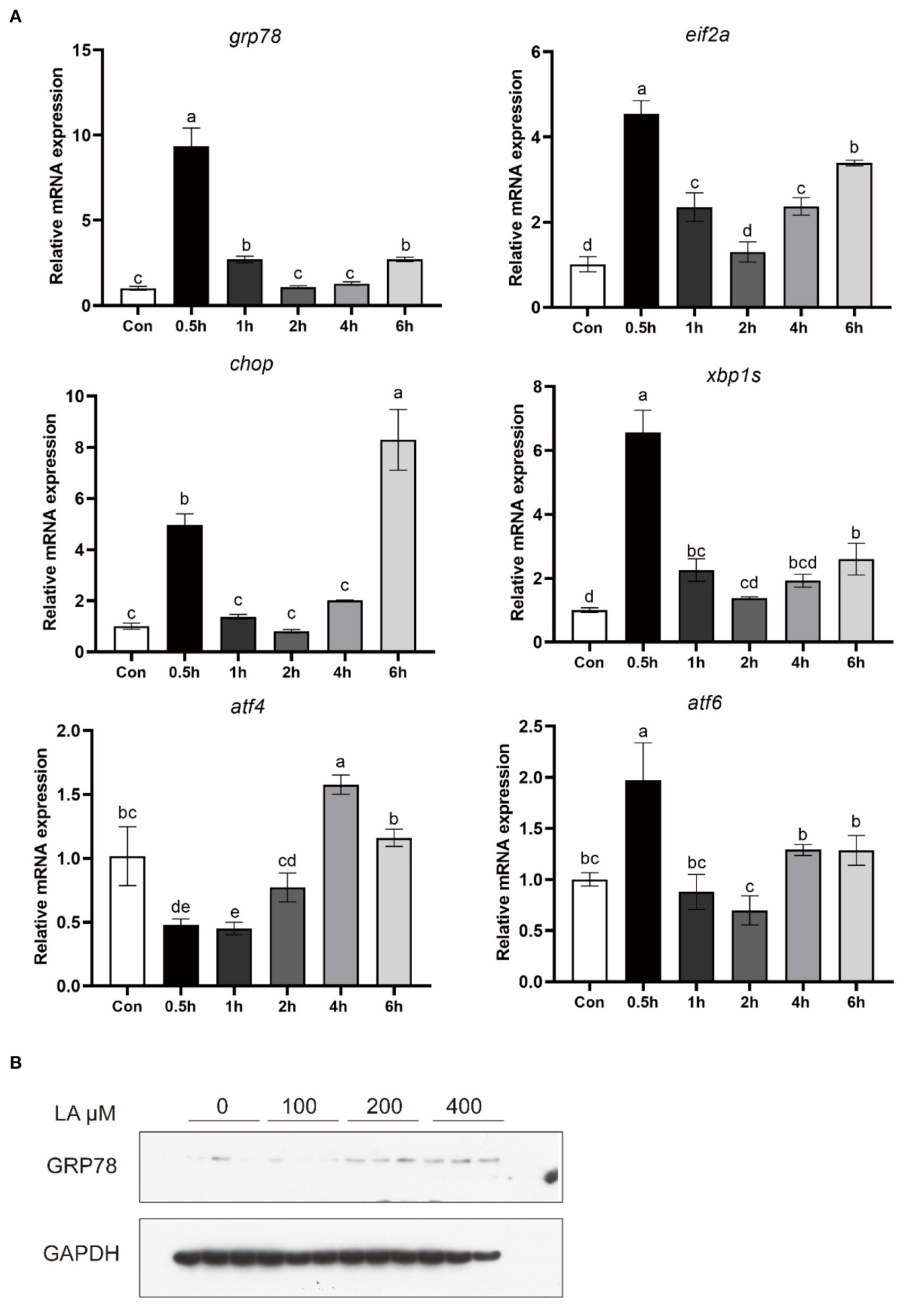
Expression of **(A)** ER stress-related genes and **(B)** GRP78 protein levels in croaker macrophages treated with LA. The significance was analyzed by one-factor ANOVA and Duncan's Multiple Range Test (means ± SEMs; *n* = 3). Means without the same label were considered different, *P* < 0.05.

To investigate the effect of LA on ER stress, macrophages were treated with LA. The mRNA level of GRP78, EIF2α, CHOP, XBP1S, ATF4, and ATF6 was significantly increased in macrophages after LA treatment (*P* < 0.05) (Figure 2A). Additionally, in croaker macrophages, increased protein levels of GRP78 were observed following LA treatment (*P* < 0.05) (Figure 2B).

### TM treatment increased pro-inflammatory gene expression

**Figure 3:**
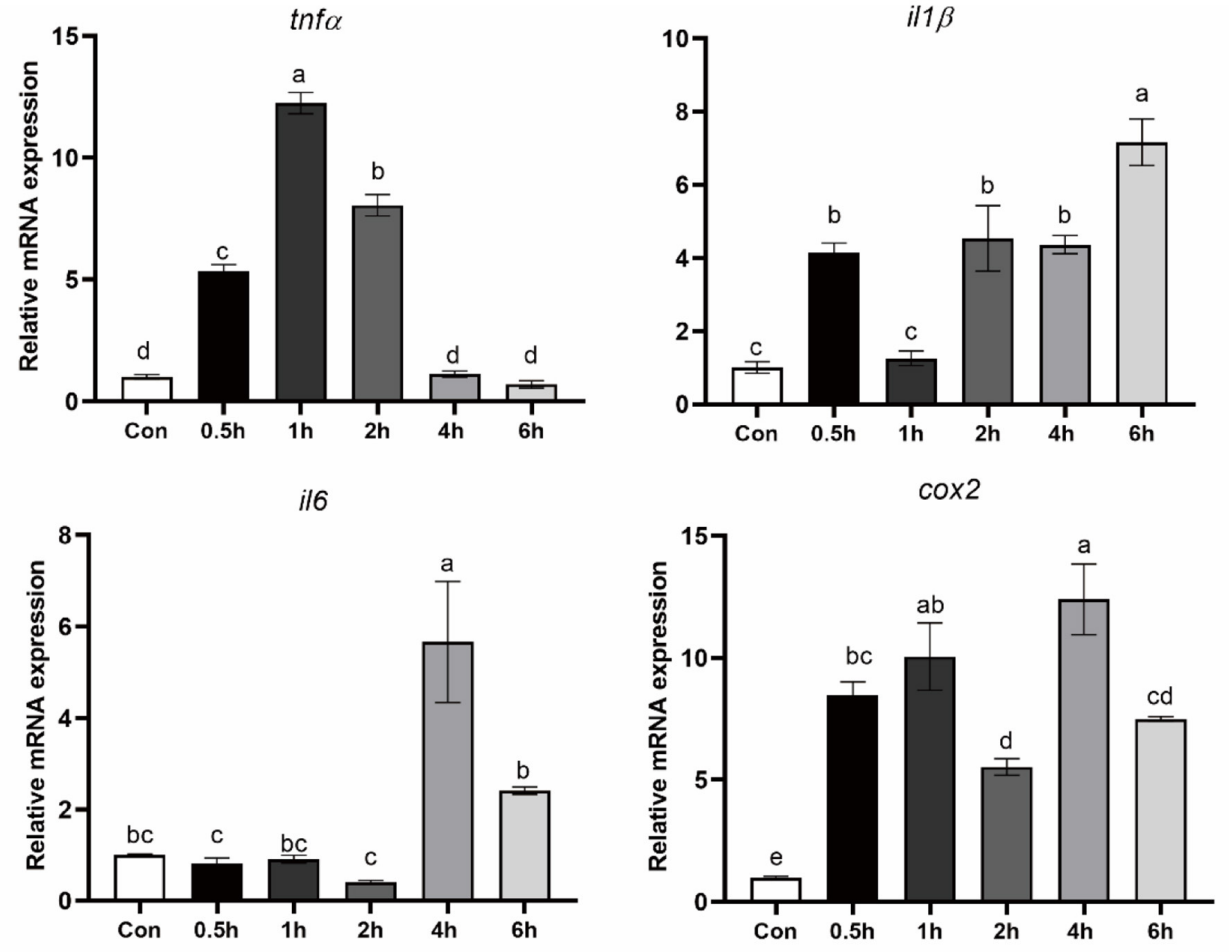
Expression of pro-inflammatory genes in croaker macrophages after treatment with TM. The significance was analyzed by one-factor ANOVA and Duncan's Multiple Range Test (means ± SEMs; *n* = 3). Means without the same label were considered different, *P* < 0.05.

To confirm the effect of activation of ER stress on inflammation, macrophages were treated with an ER stress inducer TM for 0.5, 1, 2, 4, and 6 hours. Following 0.5, 1 and 2 hours of TM treatment, TNFα expression was significantly increased (*P* < 0.05) (Figure 3). In addition, the mRNA level of IL1β, IL16, and COX2 was significantly induced after TM treatment for 4 and 6 hours (*P* < 0.05) (Figure 3).

### Activation of FXR suppressed TM-induced ER stress and pro-inflammatory genes expression

**Figure 4:**
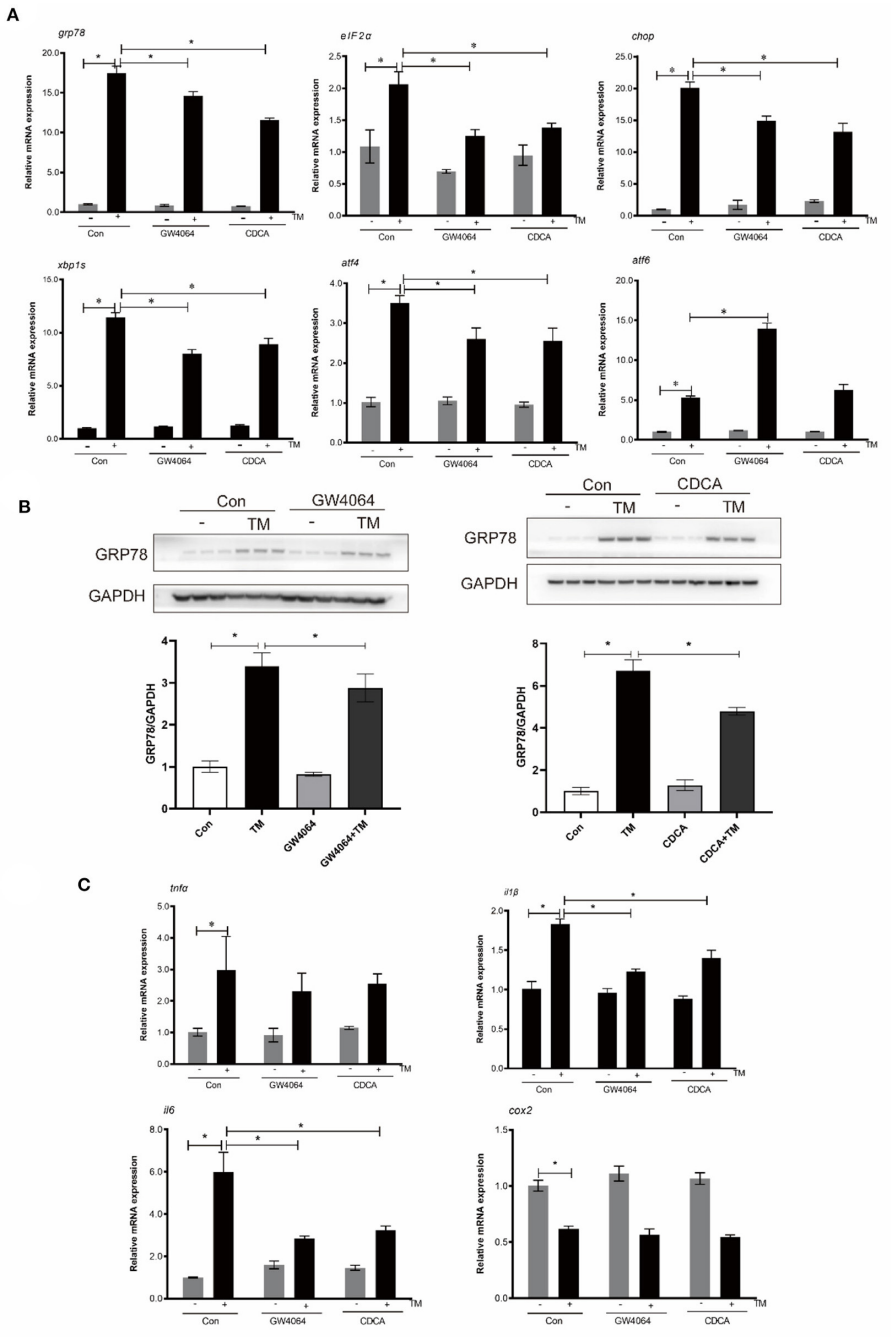
Effects of activation of FXR by ligands on TM-induced ER stress and inflammation in croaker macrophages. **(A)** Gene expression and **(B)** protein level of ER-stress markers in macrophages after TM stimulation alone or with FXR agonists (GW4064 and CDCA). **(C)** Gene expression of pro-inflammatory genes in in macrophages after TM stimulation alone or with FXR agonists. *Different between two groups; *P* < 0.05 (means ± SEMs; *n* = 3; Student's *t*-test).

In order to investigate FXR's influence on ER-induced inflammation, macrophages were treated with TM and FXR agonists (GW4064 and CDCA). The mRNA levels of GRP78, EIF2α, CHOP, XBP1S, and ATF4 were significantly decreased with GW4064 and CDCA treatment in comparison to TM alone (*P* < 0.05) (Figure 4A). Compared with the control group, TM treatment significantly increased the protein level of GRP78. Compared with the cells treated with TM alone, the protein level of GRP78 was significantly lower in cells after GW4064 or CDCA treatment (*P* < 0.05) (Figure 4B). Treatment with GW4064 or CDCA also significantly inhibited the TM-induced gene expression of IL1β and IL6 (*P* < 0.05) (Figure 4C).

### FXR overexpression suppressed TM-induced ER stress and pro-inflammatory genes expression

**Figure 5:**
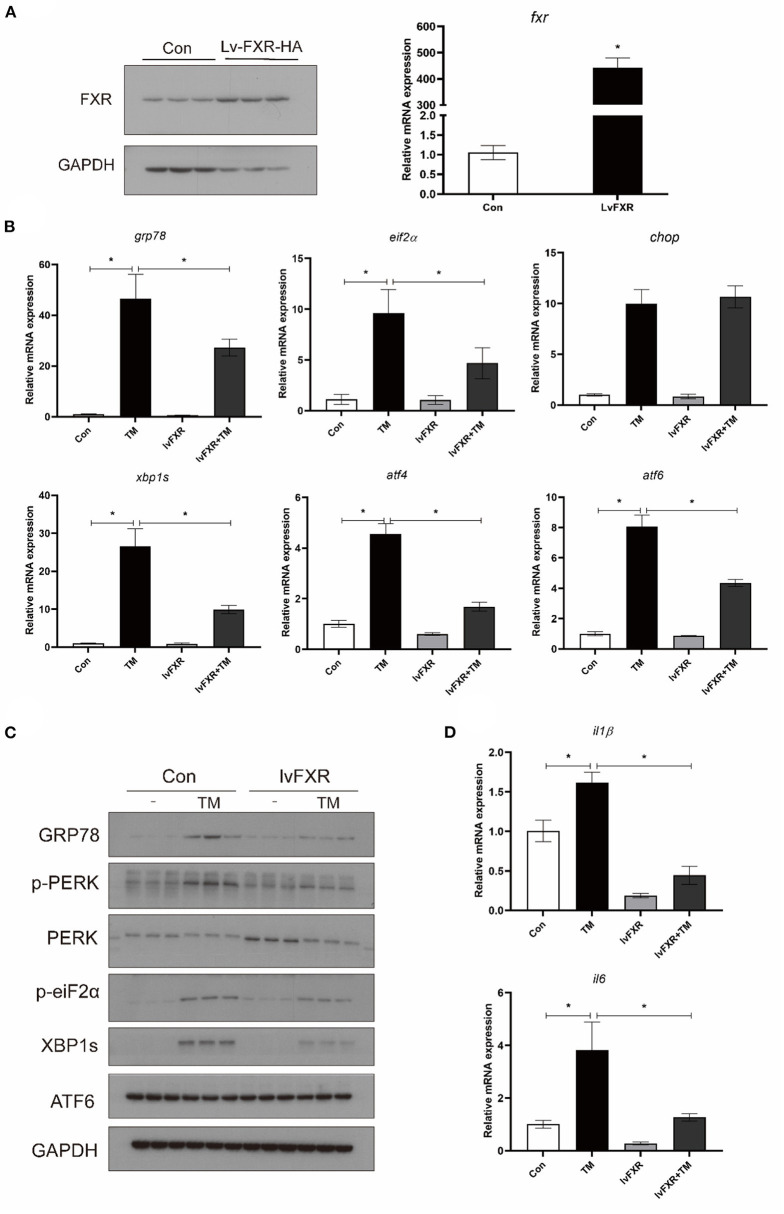
Effects of **(A)** FXR overexpression on TM-induced ER stress and inflammation in croaker macrophages. **(B)** Gene expression and **(C)** protein level of ER-stress markers in stably FXR-transfected macrophages after TM treatment. **(D)** Expression of IL1β and IL6 in stably FXR-transfected macrophages after TM treatment. *Different between two groups; *P* < 0.05 (means ± SEMs; *n* = 3; Student's *t*-test).

Croaker FXR was overexpressed in macrophages stably transfected with the FXR (LvFXR group) (Figure 5A). Compared with that in the control group, FXR overexpression had no significant impact on the gene expression of GRP78, EIF2α, CHOP, XBP1S ATF4, and ATF6 (*P* > 0.05) (Figure 5B). However, the overexpression of FXR significantly decreased the TM-induced gene expression of GRP78, EIF2α, XBP1S, ATF4, and ATF6, and phosphorylation of PERK, phosphorylation of EIF2α, and protein levels of GRP78 and XBP1s (*P* < 0.05) (Figures 5B,C). In addition, the induction of the expression of IL1β and IL6 by TM was significantly inhibited by the overexpression of FXR (*P* < 0.05) (Figure 5D).

### Knocking down FXR Promoted TM-induced ER stress and pro-inflammatory genes expression

**Figure 6:**
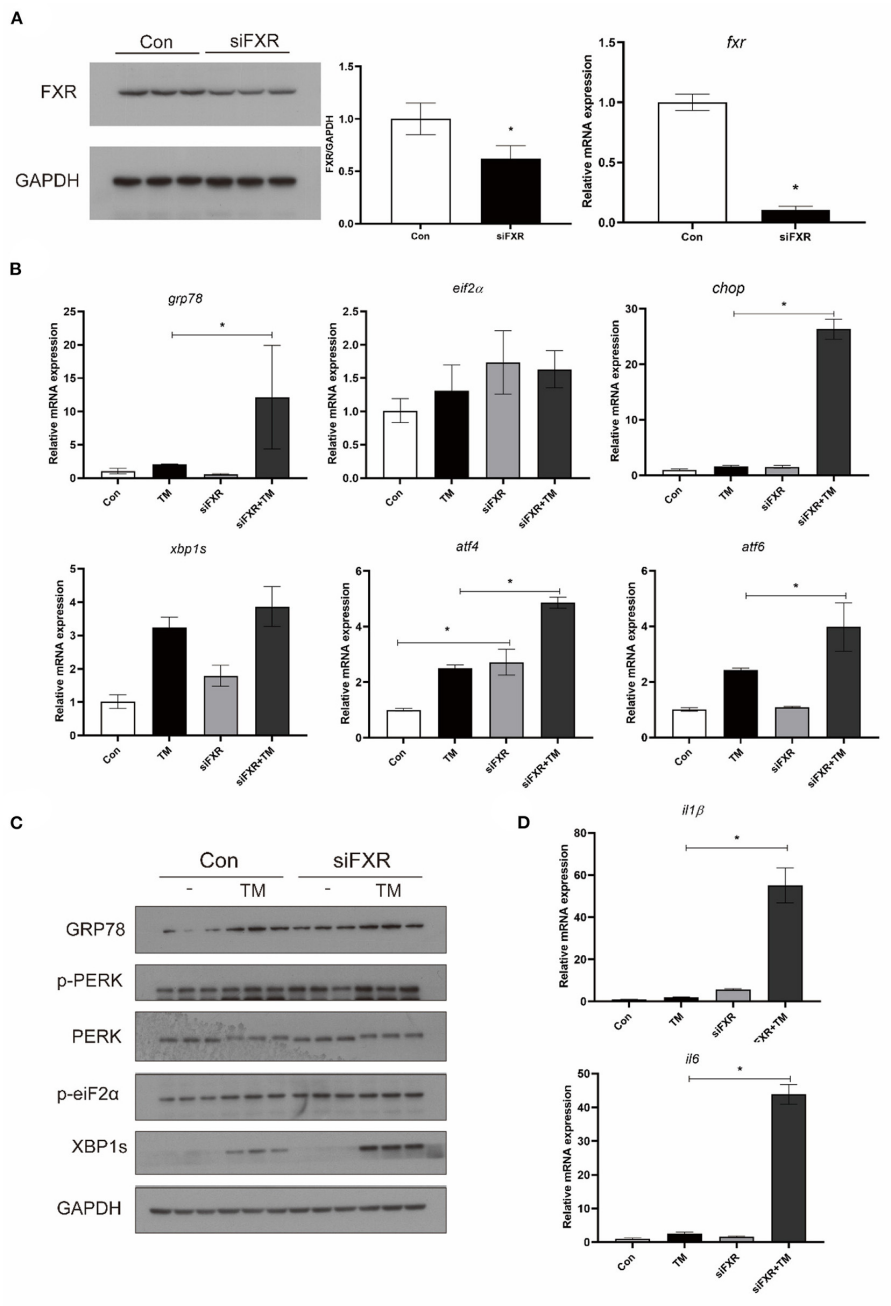
Effects of **(A)** FXR knockdown on TM-induced ER stress and inflammation in croaker macrophages. **(B)** Gene expression and **(C)** protein level of ER-stress markers in macrophages with stable interference for FXR after TM treatment. **(D)** Expression of IL1β and IL6 in macrophages with stable interference for FXR after TM treatment. *Different between two groups; *P* < 0.05 (means ± SEMs; *n* = 3; Student's *t*-test).

The expression of FXR was knocked down in macrophages with stable interference for FXR (siFXR group) (Figure 6A). The siFXR group significantly showed higher levels of ATF4 mRNA, GRP78 protein, and phosphorylated EIF2α compared with the control group (*P* < 0.05) (Figure 6B). The knockdown of FXR significantly enhanced the mRNA levels (GRP78, CHOP, ATF4, AND ATF6) and protein levels (phosphorylated PERK, phosphorylated EIF2α, GRP78, and XBP1s) induced by TM in croaker macrophages (*P* < 0.05) (Figures 6B,C). Additionally, as a result of FXR knockdown, gene expression of IL16 and IL1β were significantly enhanced in macrophages induced by TM (*P* < 0.05) (Figure 6D).

### NCK1 was involved in the regulation of PERK in the large yellow croaker

**Figure 7:**
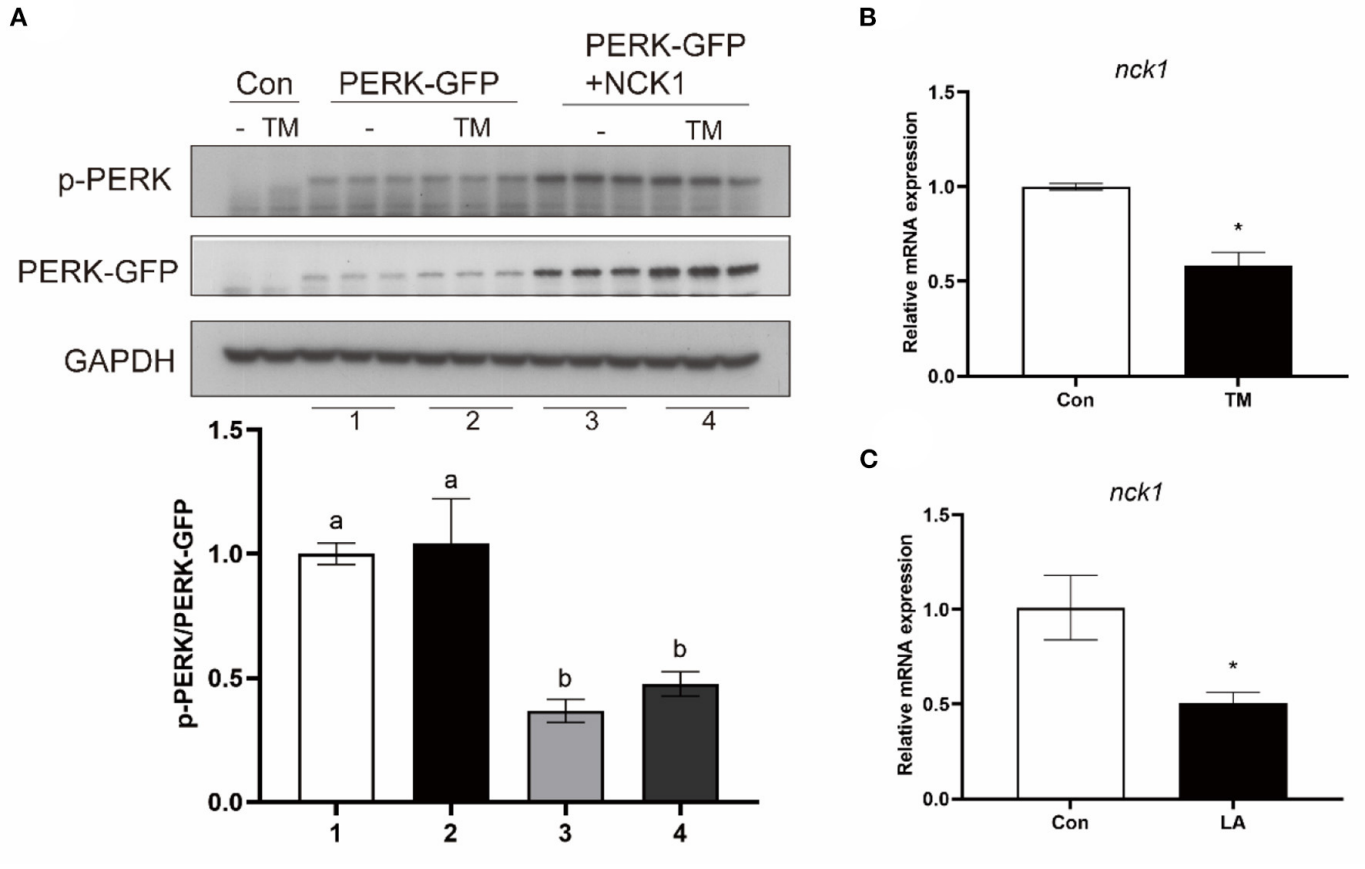
Effects of NCK1 on PERK in large yellow croakers. **(A)** Phosphorylated PERK immunoblot in HEK 293T cells after NCK1 overexpression. **(B,C)** NCK1 expression in macrophages after treatment with TM and LA. The significance was analyzed by one-factor ANOVA and Duncan's Multiple Range Test (means ± SEMs; *n* = 3). Means without the same label were considered different, *P* < 0.05. *Different between two groups (Student's *t*-test).

To investigate the effect of croaker NCK1 on PERK, HEK 293T cells were transfected with croaker NCK1 and PERK-GFP plasmids. When NCK1 and PERK-GFP plasmids were co-transfected into HEK 293T cells, PERK phosphorylation was significantly lower than in cells only transfected with PERK-GFP plasmid (*P* < 0.05) (Figure 7A). Further, NCK1 expression in macrophages was significantly reduced after TM and LA treatment (*P* < 0.05) (Figures 7B,C).

### FXR increased NCK1 expression *via* YY1 and P65

**Figure 8:**
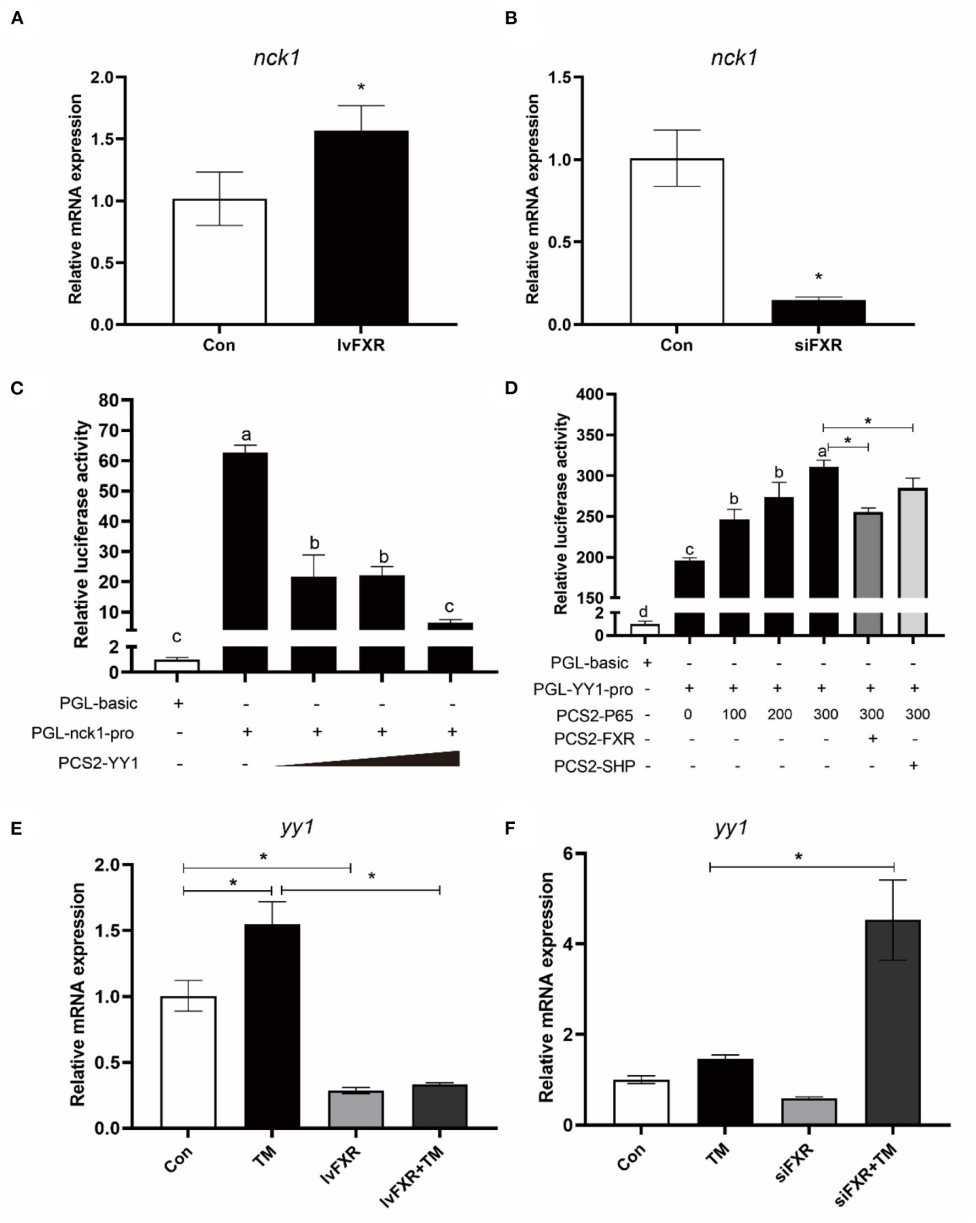
FXR regulated the expression of NCK1 *via* YY1. Effects of **(A)** FXR overexpression or **(B)** knockdown on NCK1 expression in croaker macrophages. **(C)** Relative luciferase activities of croaker NCK1 promoter in HEK 293T cells after overexpression of YY1. **(D)** Relative luciferase activities of croaker YY promoter in HEK 293T cells after overexpression of P65 and FXR. **(E,F)** Effects of FXR overexpression or knockdown on the TM-induced expression of YY1 in croaker macrophages. The significance was analyzed by one-factor ANOVA and Duncan's Multiple Range Test (means ± SEMs; *n* = 3). Means without the same label were considered different, *P* < 0.05. *Different between two groups (Student's *t*-test).

The gene expression of NCK1 was significantly higher in the stably FXR-transfected macrophages than in the control group, and it was decreased by FXR knockdown (*P* < 0.05) (Figures 8A,B). In the luciferase reporter assay, croaker YY1 overexpression inhibited NCK1 promoter activity significantly (*P* < 0.05) (Figure 8C). Moreover, croaker FXR and SHP overexpression significantly inhibited the promoter activity of YY1 induced by P65 in HEK 293T cells (*P* < 0.05) (Figure 8D). Treatment with TM significantly increased YY1 expression in macrophages, which was suppressed by FXR overexpression and enhanced by knockdown of FXR (*P* < 0.05) (Figures 8E,F).

## Discussion

Excessive and sustained inflammation and ER stress will damage the health of the body, which needs to be tightly regulated. The vicious circle that exists between ER stress and inflammation could aggravate the disease and increases the difficulty of regulation. Studies in mammals and fish showed that inflammation and ER stress could be induced by high dietary levels of vegetable oils (6, 7, 14, 33) and activation of ER stress could induce inflammation (12, 13, 15). Our previous study showed that high dietary levels of soybean oil which is rich in linoleic acid (LA) could cause inflammation in the liver, head kidney, intestine and spleen of large yellow croakers (8, 22). The present study found that high dietary levels of soybean oil and LA treatment induced ER-stress markers in head kidney of croakers and macrophages. These results suggested that high LA intake could lead to inflammation and ER stress in large yellow croakers. Based on these findings, we investigated whether ER stress regulates inflammation and how ER stress is regulated by FXR. Results showed that the ER stress inducer TM significantly increased expression of pro-inflammatory genes such as TNFα, IL1β, IL6 and COX2. Meanwhile, FXR agonist CDCA supplementation decreased SO-diet-induced ER stress *in vivo*, while croaker FXR activation by ligands reduced TM-induced ER stress and inflammation in macrophages. It appears that croaker FXR may regulate ER-stress response in fish.

In order to understand FXR's role in ER stress regulation, we established macrophages stably transfected for FXR overexpression or knockdown. By overexpressing croaker FXR, the results indicated that the TM-induced expression of ER stress markers was decreased, and the IL1β and IL16 expression were inhibited. By contrast, the knockdown of FXR significantly increased mRNA and protein levels of ER-stress-related genes and the gene expression of IL1β and IL16. In mice, FXR had an inhibitory effect on ER stress, and treating mice with FXR ligands reduced the effects of ER stress on liver cells and hepatocyte death (24). According to these results, FXR might be a promising target for reducing ER stress and inflammation in fish.

Our study explored how FXR modulated ER stress and inflammation further. All three pathways (PERK, IRE1, and ATF6) triggered by ER stress have been confirmed to be involved in inflammatory signaling (10, 11). The results of our above experiment showed that FXR overexpression inhibited the TM-induced phosphorylation of PERK and EIF2α, while the knockdown of FXR enhanced the TM-induced phosphorylation of PERK and EIF2α. These results are consistent with previous findings in mice showing that FXR activation inhibited the ER-stress-activated PERK pathway (24). According to studies in mammals, autophosphorylation activates PERK, which then phosphorylates EIF2α and increases the expression of ATF4 and CHOP (10). Through activation of the PERK-EIF2α pathway, inflammation can be induced by activating the NF-κB pathway (34–36). Meanwhile, the direct binding of ATF4 to IL6 increases inflammation (37). Previous research in large yellow croakers found that ATF4 or CHOP overexpression could increase the promoter activity of TNFα, IL1β, or COX2 (15). In addition, the present results showed that FXR overexpression inhibited the TM-induced protein levels of XBP1s which was enhanced by the knockdown of FXR. The IRE1α-XBP1s pathway has been shown to participate in the regulation of inflammation (10, 38). According to these results, the activation of FXR may regulate inflammation and ER stress by affecting the PERK and IRE1α pathways in fish.

After elucidating the effect of croaker FXR on the PERK and IRE1α pathway, we investigated the underlying mechanism. Studies in mice showed that FXR could affect the expression of NCK1 which is a negative regulator of ER stress in mammals (24, 39, 40). NCK1 affects the activation of PERK and IRE1α through its interaction with them (39–41). However, a study in obese mice showed that deletion of NCK1 attenuates hepatic ER stress signaling (42). Then, we further confirmed the effect of croaker NCK1 on PERK pathway in the present study. The results showed that overexpression of croaker NCK1 in HEK 293T cells decreased the phosphorylation of croaker PERK. In croaker macrophages, the gene expression of NCK1 was decreased after TM and LA treatment or knock-down of FXR, while FXR overexpression increased the NCK1 expression. These results indicate that FXR may affect the PERK pathway by regulating the expression of NCK1. Then, we further explored how FXR affected NCK1 expression. FXR frequently functions as a transcription factor. However, an FXR binding site was not predicted in the croaker NCK1 promoter sequence. By contrast, several binding sites for YY1, which is recognized as a transcriptional repressor protein (43), were found in the croaker NCK1 promoter. In croakers, the activity of NCK1 promoter was decreased by overexpression of YY1. Previous studies in mammals have demonstrated that nuclear factor-kB P65 induced the expression of YY1 (44). Our previous research has shown that croaker FXR and its target gene SHP can inhibit P65 transcriptional activity *via* protein interaction (22). This study shows that overexpression of croaker FXR and SHP inhibited the activity of the YY1 promoter induced by P65. Moreover, FXR overexpression suppressed the TM-induced gene expression of YY1, while the knockdown of FXR enhanced Tm-induced YY1 expression. Therefore, croaker FXR may enhance NCK1 expression by suppressing P65-induced YY1 expression, which then inhibits the PERK pathway.

In conclusion, we found that FXR was a regulator of the ER-stress response in large yellow croakers. In response to FXR activation, YY1 expression is suppressed and NCK1 expression is increased, thereby inhibiting the PERK pathway. This study contributes to enriching the knowledge of FXR as a potential target for regulating ER stress and inflammation.

## Data availability statement

The raw data supporting the conclusions of this article will be made available by the authors, without undue reservation.

## Ethics statement

The animal study was reviewed and approved by Animal Ethics Committee of Ocean University of China.

## Author contributions

JD, QA, and KM designed the research. The experiments were conducted by JD. With the help of JZ, XX, DX, and KC. Data analysis and article writing were done by JD and QA. Final approval of the manuscript was given by all authors.

## Funding

Support for this study was provided by the National Natural Science Foundation of China (Grant No. 32002398), the Key Program of National Natural Science Foundation of China (Grant No. 31830103), and the Scientific and Technological Innovation of Blue Granary (Grant No. 2018YFD0900402).

## Conflict of interest

The authors declare that the research was conducted in the absence of any commercial or financial relationships that could be construed as a potential conflict of interest.

## Publisher's note

All claims expressed in this article are solely those of the authors and do not necessarily represent those of their affiliated organizations, or those of the publisher, the editors and the reviewers. Any product that may be evaluated in this article, or claim that may be made by its manufacturer, is not guaranteed or endorsed by the publisher.
