# Supplementary material for: Activation of farnesoid X receptor suppresses ER stress and inflammation via the YY1/NCK1/PERK pathway in large yellow croaker (Larimichthys crocea)
Source: Front Nutr. 2022 Nov 24;9:1024631. doi: 10.3389/fnut.2022.1024631 (PMC9731767; doi:10.3389/fnut.2022.1024631)
Supplement: Supplementary file 1 [file Table_1.DOCX]

**SUPPLEMENTAL TABLE 1.** Formulation and proximate analysis of the experimental diets^1^(Du et al., 2017).

|  | Diets | | | | |
| --- | --- | --- | --- | --- | --- |
| Ingredients g/kg | | FO | SO | SO-CDCA 300 | SO-CDCA 900 |
| Fish meal | | 390 | 390 | 390 | 390 |
| Soybean meal | | 200 | 200 | 200 | 200 |
| Wheat meal | | 230 | 230 | 230 | 230 |
| Wheat starch | | 60 | 60 | 60 | 60 |
| Fish oil | | 60 | 0 | 0 | 0 |
| Soybean oil | | 0 | 60 | 60 | 60 |
| Soybean lecithin | | 15 | 15 | 15 | 15 |
| Vitamin premix^2^ | | 20 | 20 | 20 | 20 |
| Mineral premix^3^ | | 20 | 20 | 20 | 20 |
| Attractant^4^ | | 3 | 3 | 3 | 3 |
| Mold inhibitor^5^ | | 1 | 1 | 1 | 1 |
| CDCA | |  |  | 0.3 | 0.9 |
| Proximate composition (% dry matter) | | | |  |  |
| Crude protein | | 45.2 | 46.1 | 45.7 | 45.6 |
| Crude lipid | | 11.5 | 11.4 | 10.8 | 11.1 |

^1^ CDCA, chenodeoxycholic acid; FO, fish oil; SO, soybean oil; SO-CDCA 300, soybean oil diet supplemented with 300 mg/kg CDCA; SO-CDCA 900, soybean oil diet supplemented with 900 mg/kg CDCA.

^2^Vitamin premix (mg or g/kg diet): cholecalciferol, 5 mg; retinol acetate, 32 mg; thiamin 25 mg; cyanocobalamin, 10 mg; riboflavin, 45 mg; pyridoxine HCl, 20 mg; ascorbic acid, 2000 mg; alpha-tocopherol (50%), 240 mg; menadione, 10 mg; pantothenic acid, 60 mg; inositol, 800 mg; niacin acid, 200 mg; folic acid, 20 mg; biotin (2%), 60 mg; choline chloride (50%), 4000 mg; microcrystalline cellulose, 12.47 g. ^3^Mineral premix (mg or g/kg diet): CuSO_4_·5H_2_O, 10 mg; Ca (IO_3_)_2_·6H_2_O (1%), 60 mg; CoCl_2_·6H_2_O (1%), 50 mg; FeSO_4_·H_2_O, 80 mg; MgSO_4_·7H_2_O, 1200 mg; MnSO_4_·H_2_O, 45 mg; NaSeSO_3_·5H_2_O (1%), 20 mg; ZnSO_4_·H_2_O, 50 mg; CaH_2_PO_4_·H_2_O, 10 g; Zeolite, 8.485 g. ^4^Attractants: glycine and betaine ^5^Mold inhibitor: contained 50% calcium propionic acid and 50% fumaric acid.

**SUPPLEMENTAL TABLE 2** Primers used for qPCR analysis^1^

| Genes | Forward Sequences (5′–3′) | Reverse Sequences (5′–3′) |
| --- | --- | --- |
| *fxr* | TGGAGGAAAGGATACGCAAGAGTG | TGTCAGGATGGTTACGGTGGTG |
| *grp78* | GGTGGCGATGACAAGCAAAC | CTGAGAACAGCAGCAACAAGC |
| *eif2α* | ATGCCGGGGCTCAGCTGTC | CTAGTCCTCTGCTTTGGCCTCC |
| *xbp1s* | GTCTTCTGAGTCCGCAGCAGGTG | AGGATGTCCAGAATGCCCAGTAG |
| *chop* | TCTGGATGTTCTGGAGAGTTGTTC | AGGATGATGATGAGGTGTGATGC |
| *atf4* | GCCGTTATTCTGCTCCATCTTCT | AGACCTTACCCTGAGCCCACAT |
| *atf6* | CAGATAATAAGGAGGCTGAGAGTGC | CGTAGGTATGATGAGGTGCGTAGT |
| *cox2* | CTGGAAAGGCAACACAAGC | CGGTGAGAGTCAGGGACAT |
| *il-1β* | AGCCAATCTGGCAAGGATCA | GCTGATGAACCAGTTGTTGT |
| *il6* | CGACACACCCACTATTTACAAC | TCCCATTTTCTGAACTGCCTCT |
| *tnf**a* | CGTCCTGGTGTTTGCTTGGT | TGTTTTCTCGGCAGTCGTCTT |
| *shp* | GCGACGGACAGTGTGCTTGAA | ACTGGTCGTTTGGTGGCATCTG |
| *nck1* | GCAAGAAAGCGGGCAAAAAG | GCCCACATCGTGACAGAAAAC |
| *yy1* | TCACACACGCCAAAGCCAAA | ACAAAGGGAATCTCACAACAAAGC |
| *gapdh* | GACAACGAGTTCGGATACAGC | CAGTTGATTGGCTTGTTTGG |
| *β-actin* | CTACGAGGGTTATGCCCTGCC | TGAAGGAGTAACCGCGCTCTG |

*cox2*, cyclooxygenase 2; *il-1β*, interleukin 1 beta; *il6*, interleukin 6; *shp*, small heterodimer partner; *gapdh*, glyceraldehyde-3-phosphate dehydrogenase; *tnfa*, tumor necrosis factor alpha; *atf4,* activating transcription factor 4*; atf6,* activating transcription factor 6*; chop,* C/EBP homologous protein*; fxr*, farnesoid X receptor; *eif2α,* eukaryotic initiation factor 2α*; grp78,* glucose-regulated protein 78*; xbp1s,* X-box-binding protein 1; *nck1*, non-catalytic region of tyrosine kinase adaptor protein 1; *yy1*, Yin-Yang 1.
